# Supplementary material for: A neurocognitive mechanism for increased cooperation during group formation
Source: Commun Psychol. 2024 Dec 23;2:127. doi: 10.1038/s44271-024-00177-3 (PMC11666775; doi:10.1038/s44271-024-00177-3)
Supplement: Supplementary file 2 — Supplementary Materials [file 44271_2024_177_MOESM2_ESM.pdf]

Supplementary Materials

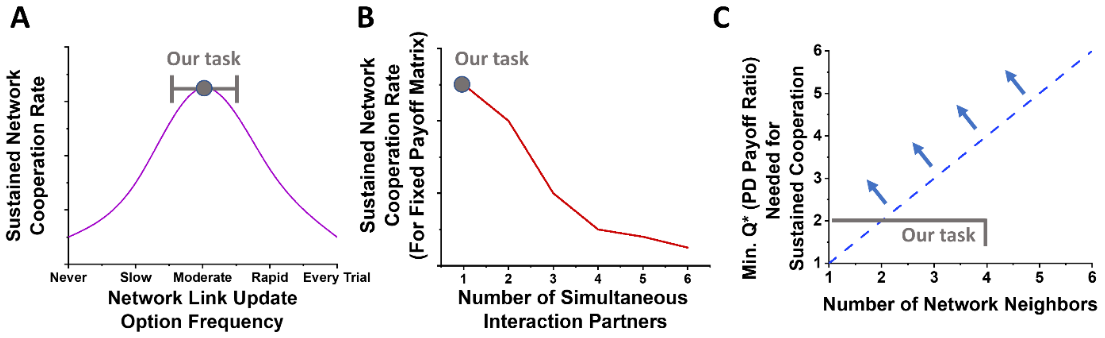

Figure S1. Motivation for iterative embedded-dyad network prisoner's dilemma design

(A) Sketch conceptually summarizing prior results suggesting dynamic link variants of network prisoner's dilemma (PD) foster cooperation in iterative tasks above static link variants, as long as the link update frequency is not too frequent[1]. We chose a moderate update frequency for both newcomer and link break options (Methods). (B) Sketch conceptually summarizing prior results finding for larger group sizes and fixed payoff matrix, that embedded dyadic PD interactions foster cooperation above simultaneous play interactions with many neighbors at once[2–5]. (C) In typical PD notation, our task payoff matrix has T=60, R=30, P=0, and S=-30 (Methods). Rand et al. found the condition for cooperation to succeed in typical static network PD designs is Q* > k, for group size k, and Q*=(P+S-R-T)/(R+S-P-T), which we have plotted[6]. For our task Q*=(0-30-30-60)/(30-30-0-60) = 2. Our task is not static, so this plot should be interpreted as an approximate guideline. However, we have chosen payoff matrix values on the boundary of this plot to avoid biasing prosocial tendencies with unbalanced payoffs.

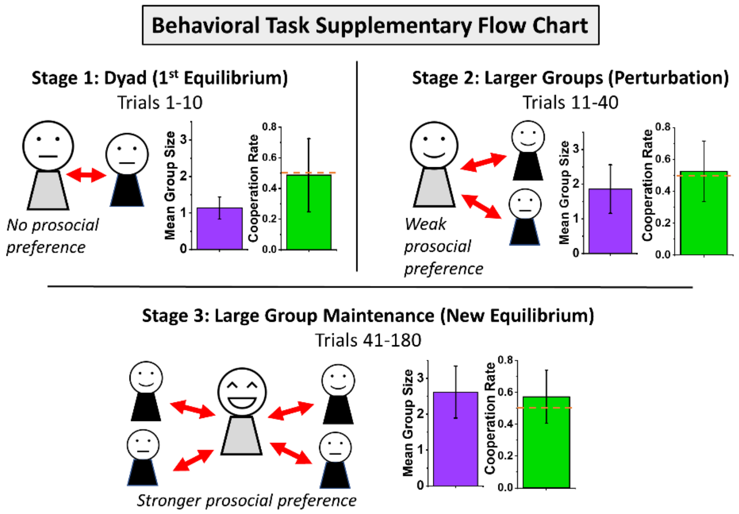

Figure S2: Additional experimental task flow information and initial cooperation rate results [related to Figure S1]

Stage 1: For approximately the first ten trials, subjects start in the typical dyad (one partner) prisoner dilemma context. The mean group size is one social partner, and mean cooperation is slightly under 50% for the first ten trials (N=83). Stage 2: Over approximately the next few tens of trials, subjects generally experience larger group sizes of 2-5 social partners, with a mean group size of two social partners and mean cooperation rate of slightly over 50% now for trials 11-40 (N=75). Stage 3: Throughout the rest of the experiment, subjects display more of a “group maintenance mindset” as they dynamically move through the full range of group sizes. The mean group size is near three social partners, and the mean cooperation approaches 60% (N=75). All error bars are SD. The red dotted line on the cooperation rate plots marks 50%.

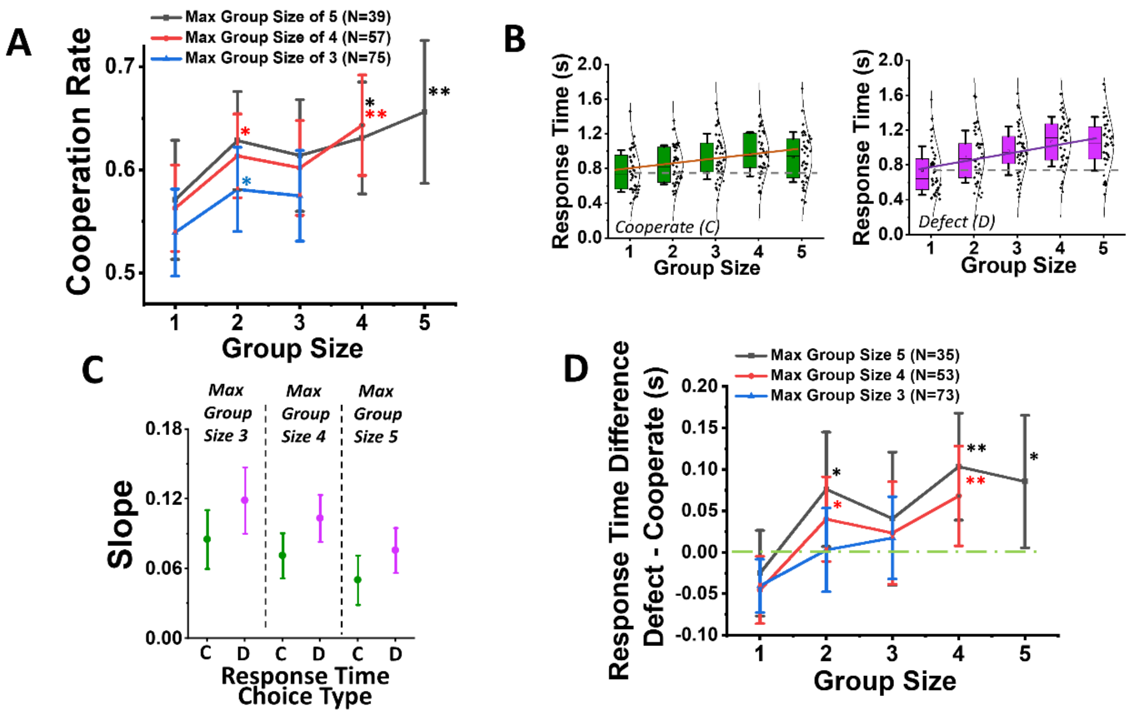

Figure S3. Group size effects aggregated by maximum group size.

(A) Between-subject mean cooperation rate (CR) for all subject sampling subsets based on the maximum group size reached in each subject's session. (B) Plots of the mean response time (RT) per group size, of each subject who had a max group size of 5 (N = 39), as an example to show the underlying distribution of the RT data, with cooperate RTs (left) plotted separately from defect RTs (right). Box plots are 25%/75% box boundaries and 1 SD whisker. Slopes of these plots were calculated for each sampling subset, and plotted in (C) with the same color scheme for cooperation (green) and defect (pink) as (B). Error bars in (C) are 95% CI. (D) RT differences for defect minus cooperate, plotted against group size. In (A) and (D) error bars are 95% CI. Statistical significance was calculated through Holm-Bonferroni-corrected pairwise t-tests relative to the group size of 1, after one-way repeated measures ANOVA, with * being for $p < 0.05$ and ** for $p < 0.01$. (Table S1 and S2)

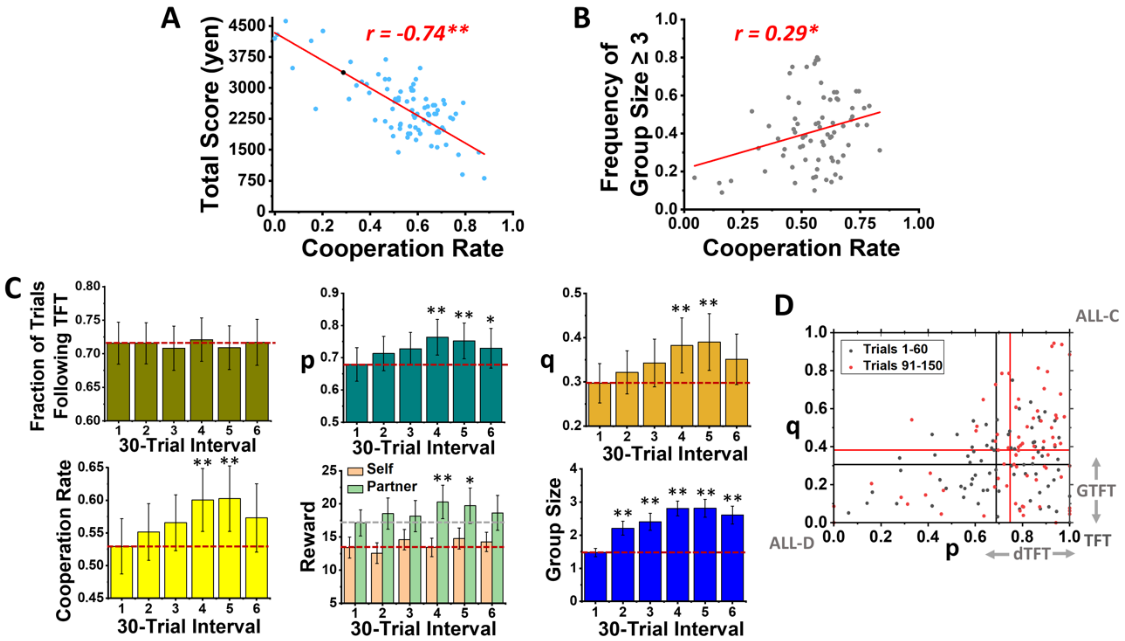

Figure S4. Subject behavioral strategy analysis

(A) Cumulative score versus mean cooperation rate for all subjects (N=87, Methods). (B) Plot showing how cooperation rate correlates with the frequency of being in a group size of three or higher (N=75). Pearson correlation coefficients are presented in red text. (C) Plots for 30 trial intervals across the 180-trial session of (left-to-right, top-to-bottom): the mean fraction of trials that subjects' choices follow pure tit-for-tat (TFT), the mean probability of self-cooperation after a partner cooperation (p), the mean probability of self-cooperation after a partner defection (q), the mean self-cooperation rate, the mean reward rate for the subject (orange) versus partner (green), and the mean group size. Statistical significance was calculated through Holm-Bonferroni-corrected pairwise t-tests relative to first 30 sessions, after ANOVA (N=75) (Table S3). Error bars are 95% CI. (D) Scatterplot of mean q versus p per subject (N=75) for initial trials 1-60 (black) and trials of maximum p-q change 90-150 (red), with mean value per color marked with the straight lines. Typical game theory policies are labeled on the plot for reference: TFT, always cooperate (ALL-C), always defect (ALL-D), generous TFT (GTFT), and our addition, devious TFT (dTFT). * is for $p < 0.05$ and ** for $p < 0.01$.

Table S1: Figure S4a Statistics

Pairwise Comparison (repeated measures, Holm-Bonferroni)	*Prob>|t|* (Max Group Size of 3)	*Prob>|t|* (Max Group Size of 4)	*Prob>|t|* (Max Group Size of 5)
Group size 1 and 2	0.040	0.033	0.057
Group size 1 and 3	0.080	0.100	0.154
Group size 1 and 4	-----	<0.001	0.047

Group size 1 and 5	-----	-----	0.005

ANOVA results listed in order of max group size of 3, 4, 5 respectively:

Mauchly's Test of Sphericity, Prob>ChiSq: <0.001, 0.004, 0.006

Mauchly's Test of Sphericity (Greenhouse-Geisser Epsilon): 0.849, 0.820, 0.768

Mauchly's Test of Sphericity (Huynh-Feldt Epsilon): 0.867, 0.860, 0.843

Repeated measures ANOVA within-subject, Prob>F (Sphericity-Assumed): 0.086, 0.009, 0.072

Repeated measures ANOVA within-subject, Prob>F (Greenhouse-Geisser): 0.095, 0.015, 0.091

Repeated measures ANOVA within-subject, Prob>F (Huynh-Feldt): 0.094, 0.013, 0.084

Table S2: Figure S3d Statistics

Pairwise Comparison (repeated measures, Holm-Bonferroni)	*Prob>|t|* (Max Group Size of 3)	*Prob>|t|* (Max Group Size of 4)	*Prob>|t|* (Max Group Size of 5)
Group size 1 and 2	0.148	0.018	0.028
Group size 1 and 3	0.054	0.056	0.149
Group size 1 and 4	-----	0.002	0.005
Group size 1 and 5	-----	-----	0.016

ANOVA results listed in order of max group size of 3, 4, 5 respectively:

Mauchly's Test of Sphericity, Prob>ChiSq: 0.989, 0.740, 0.739

Mauchly's Test of Sphericity (Greenhouse-Geisser Epsilon): 1.000, 0.968, 0.920

Mauchly's Test of Sphericity (Huynh-Feldt Epsilon): 1.000, 1.000, 1.000

Repeated measures ANOVA within-subject, Prob>F (Sphericity-Assumed): 0.134, 0.014, 0.045

Repeated measures ANOVA within-subject, Prob>F (Greenhouse-Geisser): 0.134, 0.015, 0.050

Repeated measures ANOVA within-subject, Prob>F (Huynh-Feldt): 0.134, 0.014, 0.045

Table S3: Figure S5c Statistics

Pairwise Comparison (repeated measures,	Fraction of Trials Following TFT, *Prob>|t|*	*"p"* , *Prob>|t|*	*"q"* , *Prob>|t|*	Cooperation Rate, *Prob>|t|*	Self Reward, *Prob>|t|*	Partner Reward, *Prob>|t|*	Group Size, *Prob>|t|*

Holm-Bonferroni)							
Interval 1 and 2	0.993	0.181	0.401	0.349	0.390	0.223	<0.001
Interval 1 and 3	0.622	0.055	0.123	0.123	0.219	0.370	<0.001
Interval 1 and 4	0.729	<0.001	0.004	0.003	0.974	0.006	<0.001
Interval 1 and 5	0.649	0.004	0.002	0.002	0.158	0.025	<0.001
Interval 1 and 6	0.941	0.048	0.068	0.064	0.371	0.186	<0.001

ANOVA results listed in order of columns (TFT, *p*, *q*, Cooperation Rate, Reward (self), Reward (partner), Group Size):

Mauchly's Test of Sphericity, Prob>ChiSq: 0.620, <0.001, <0.001, 0.009, 0.948, 0.006, <0.001

Mauchly's Test of Sphericity (Greenhouse-Geisser Epsilon): 0.943, 0.804, 0.815, 0.845, 0.964, 0.836,0.725

Mauchly's Test of Sphericity (Huynh-Feldt Epsilon): 1.000, 0.856, 0.868, 0.908, 1.000, 0.892,0.767

Repeated measures ANOVA within-subject, Prob>F (Sphericity-Assumed): 0.957, 0.018, 0.015, 0.014, 0.178, 0.089, <0.001

Repeated measures ANOVA within-subject, Prob>F (Greenhouse-Geisser): 0.951, 0.027, 0.023, 0.020, 0.181, 0.102, <0.001

Repeated measures ANOVA within-subject, Prob>F (Huynh-Feldt): 0.957, 0.024, 0.020, 0.017, 0.178, 0.098, <0.001

Behavioral patterns for participants with negative social tendency

Out of 83 participants included in the analysis, 21 exhibited a negative social tendency (*i.e.*, tended to defect more often than cooperate). Analyzing data from only these subjects, revealed no effect of group size on cooperation β = 0.005 95% CI [-0.158, 0.167] p=0.96; but a weak, yet significant negative effect of group size on reciprocity β = -0.075 95% CI [-0.150, 0.001] p=0.046. Similarly, interaction distance did not affect cooperation β = -0.010 95% CI [-0.006, 0.041] p=0.72, but significantly affected reciprocity, β = -0.2501 95% CI [-0.359, -0.141] p<0.001, consistent with the memory effect. Models used were identical for the ones used in the main analysis (Methods). Figure S5 shows these effects.

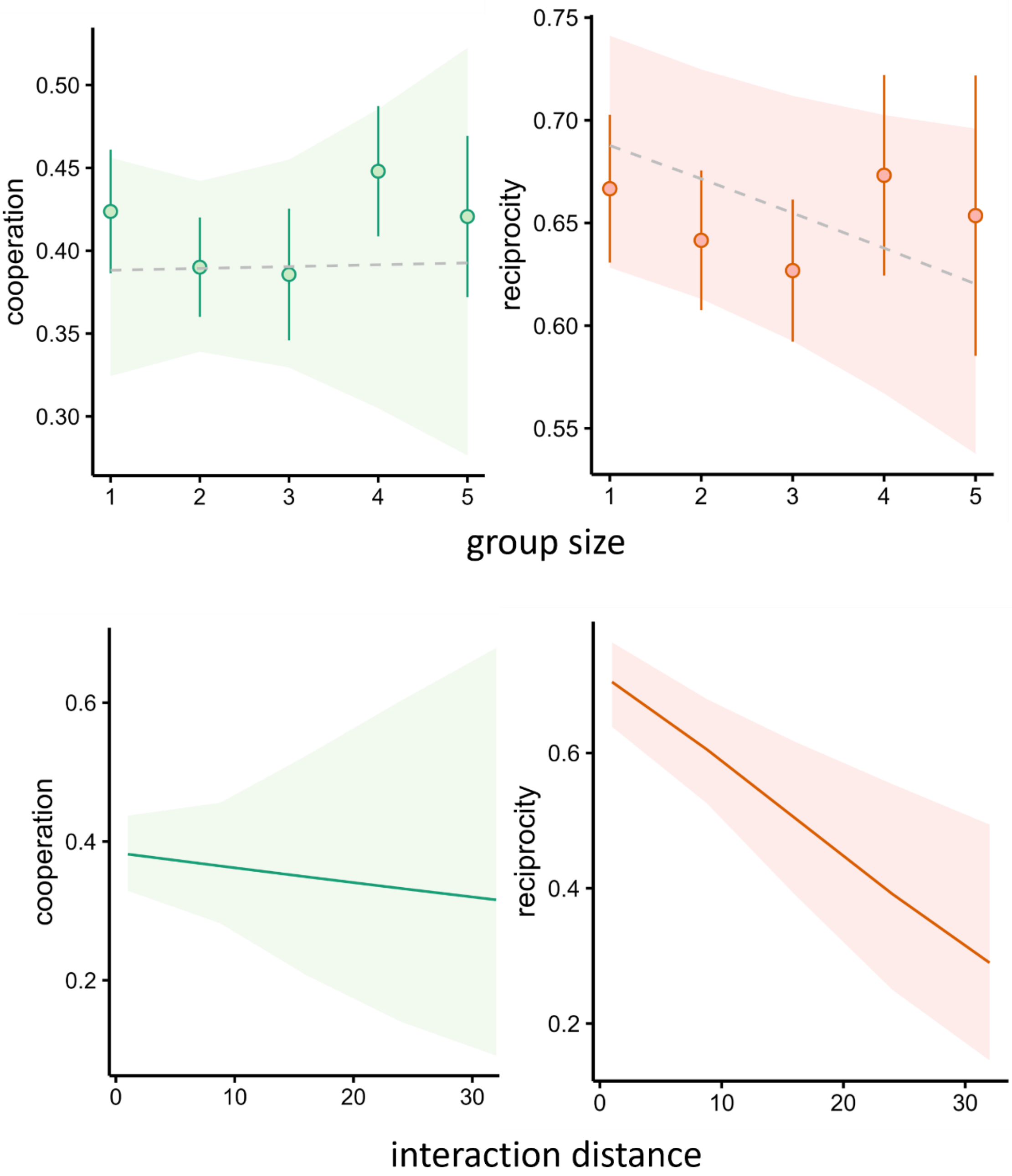

Figure S5 Behavioral patterns for participants with negative social tendency. Upper left: cooperation as a function of group size; upper right: reciprocity as a function of group size; lower left: cooperation as a function of interaction distance; lower right: reciprocity as a function of interaction distance. Error bars and ribbons represent 95% CI.

Recovery of model parameters

To test recoverability of the parameters of the winning model, we 1) sampled 10 values from posterior distributions of the group-level parameters of the fitted model 2) simulated individual-level parameters from the sampled posteriors 3) simulated experimental data from

parameter values sampled in step 2 for 83 synthetic participants, each performing 196 trials of the task 4) Fitted the data simulated in step 3 using the winning model. Figure S6 shows the group-level posterior estimate vs *true* value (simulated in step 2), as well as shows correlations between *true* (step 3) and fitted individual-level parameter values.

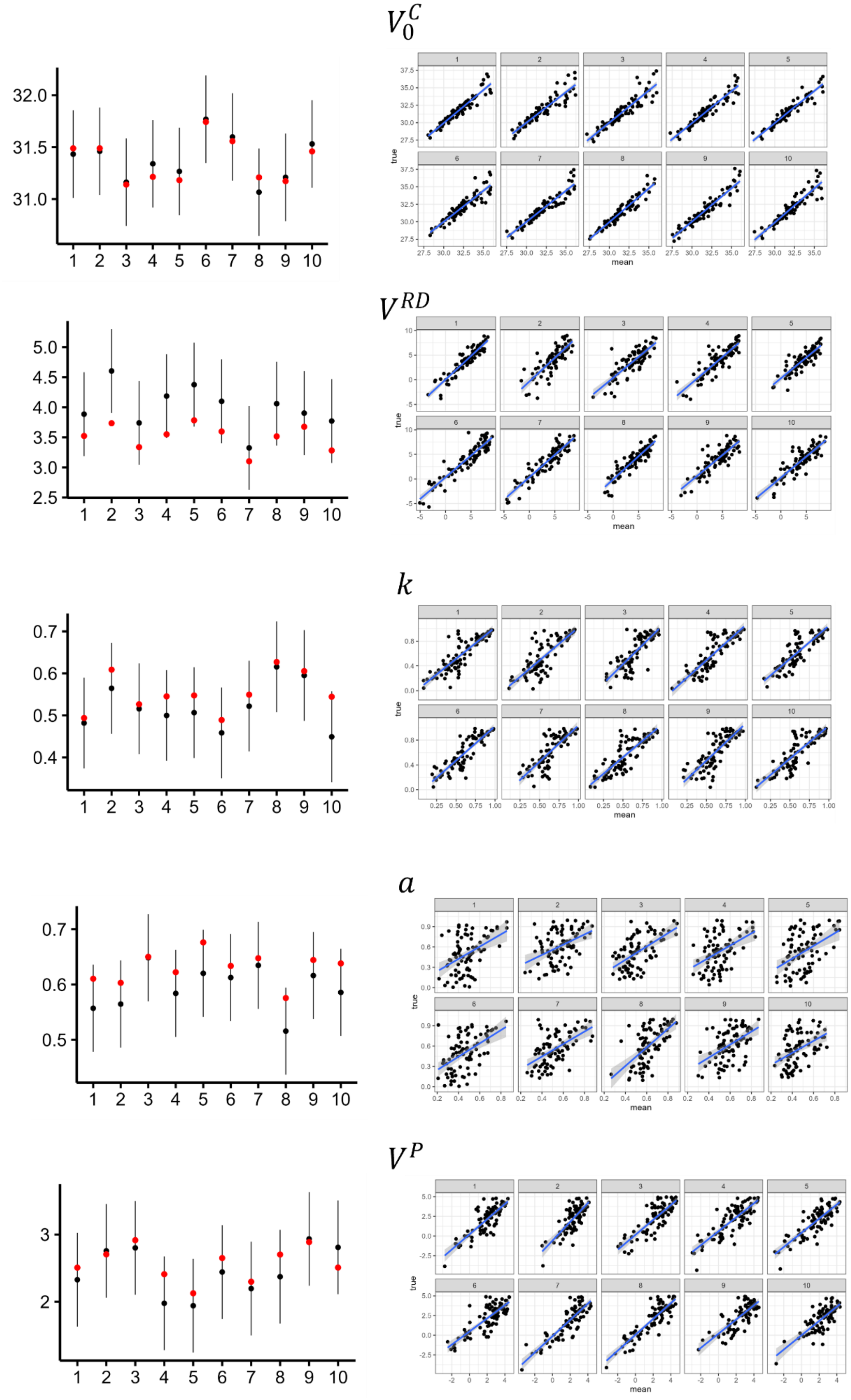
V_0^C
V^{RD}
k
a
V^P
true
mean

Figure S6 Parameter recovery. Each row represents recovery of a given parameter based on 10 simulations. Left column: group-level true values (red dots) vs 90% intervals from posterior model fit. Right column: correlations between true and fitted individual parameters. Each panel represents a single simulation.

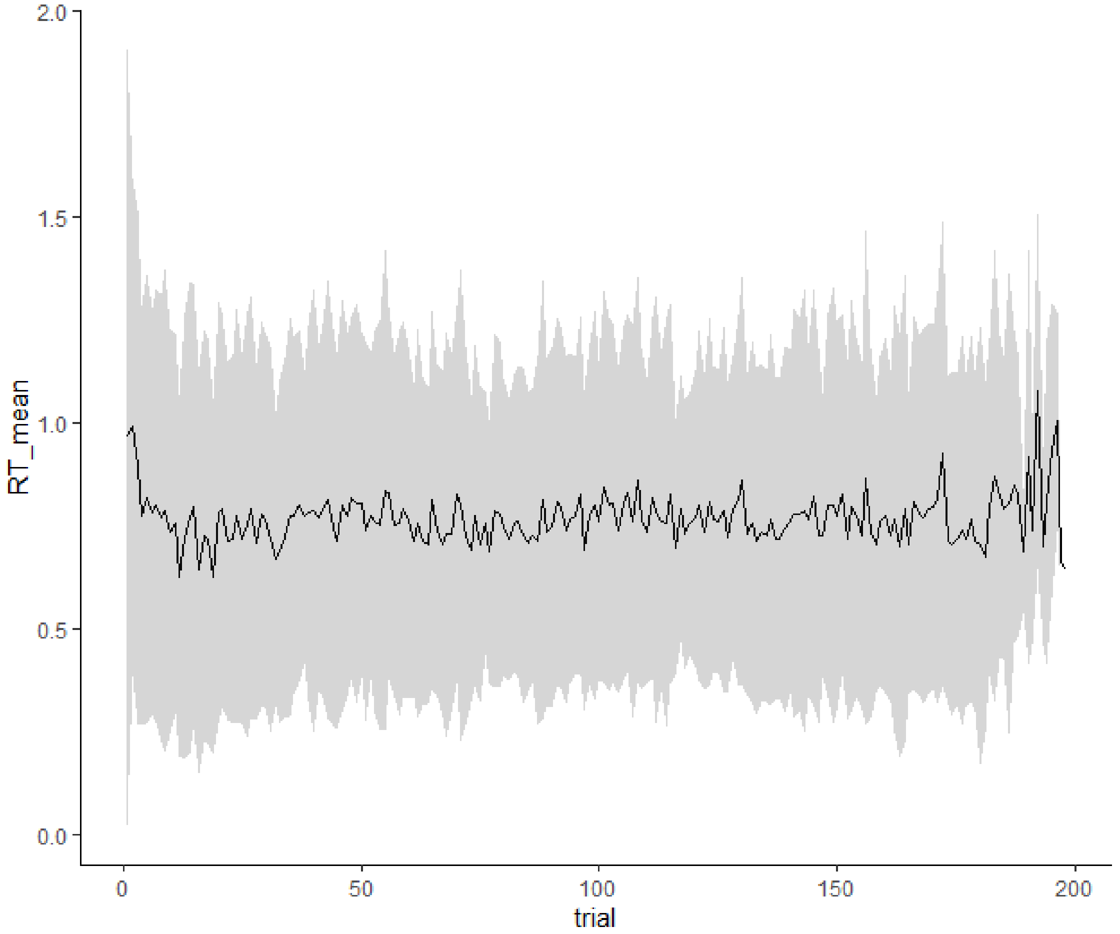

Figure S7. Effects of trial number on reaction time (RT). Black line represents mean RT at each trial averaged across all participants. Gray ribbon represents around the estimate represents +/- 1 SD).

Table S4. Localization of all whole-brain effects

Cooperation				
Structure	Peak MNI coordinates	Cluster size	Peak-level t-value	p(FWE-corr)
VS/NAcc	8 0 -6	278	6.25	p=0.003
Reciprocity				
Structure	Peak MNI coordinates	Cluster size	Peak-level t-value	p(FWE-corr)

dACC	6 22 44	957	5.88	p<0.001
right AI	24 14 -16	527	4.74	p<0.001
left AI	-32 18 -4	247	4.53	p=0.005
Forgiveness				
Structure	Peak MNI coordinates	Cluster size	Peak-level t-value	p(FWE-corr)
right MFG	32 52 10	4829	6.82	p<0.001
left Cerebellum	-49 -56 -32	5836	6.76	p<0.001
right AI	36 18 2	636	6.03	p<0.001
left IFG	-46 4 16	512	5.72	p<0.001
left SMG	-36 -32 32	564	5.71	p<0.001
right SPL	28 -50 42	1814	5.63	p<0.001
left MFG	-22 -2 44	206	5.63	p=0.001
left Pallidum	-10 0 0	205	5.39	p=0.001
left SPL	-22 -56 34	261	5.13	p=0.003
left FFG	-37 -58 -10	145	4.53	p=0.047
left MFG	-40 44 6	188	3.78	p=0.016
Betrayal				
Structure	Peak MNI coordinates	Cluster size	Peak-level t-value	p(FWE-corr)
right DLPFC	40 48 24	333	6.42	p=0.002
left DLPFC	-36 40 14	185	6.35	p=0.032
right Cerebellum	34 -60 -48	168	4.23	p=0.046
Forgetting				
Structure	MNI coordinates	Cluster size	Peak-level t-value	p(FWE-corr)
left FFG	40 -52 -22	268	5.80	p<0.001
precuneus	-12 -66 30	2177	5.52	p<0.001
right FFG	-38 -48 -22	299	5.42	p=0.001
right Cerebellum	10 -76 -38	142	5.36	p=0.047

Effects of Time

Potential concern can be raised as to whether observed changes in cooperation and reciprocity can be attributable to time. To test this, we correlated trial number with both outcome variables per participant. For cooperation, we found an average correlation of 0.026 95% CI [-0.008, 0.061], and for reciprocity, an average correlation of 0.015 95% CI [-0.01, 0.036]. We conclude that there's no evidence to suggest this might be the case.

Can the Effect be Explained by Prosocial People Having Larger Groups?

If this were to be the case, it would suggest that our proposal mechanism is merely a statistical artifact. Luckily, a careful hierarchical analysis allows for deconfounding these two explanations, by analysing between-person and within-person variance separately. This hypothesis leads to 2 clear predictions:

1. The average cooperation should correlate with average group size across participants.
2. Within individuals, group size should NOT affect cooperation rate.

The first person relates to between-person variability, while the other, to within-person effects. Both of those are easily testable given our design, and both need to necessarily be true to confirm this alternative explanation.

1. The effect average prosocial tendency on average group size is small but significant ($r=0.22$, $t(81) = 2.08$, $p=0.04$), suggesting that, unsurprisingly, more prosocial participants experience larger group sizes.
2. If we can observe the same participants expressing higher levels of cooperation in larger groups, we can be certain that there exist a causal mechanism linking increased group size with higher cooperation. This is in fact what we see in our data. To test this directly, we isolated the within-person effect by performing a mixed-effect analysis with group-centered predictors. This procedure removes the between-participant association, and all the remaining variance the model explains relates to the within person effect[7]. This analysis indicated a strong, positive within-level effect of group size on cooperation: beta=0.093, SE=0.015, $z=6.144$, $p<0.001$.

Together, these two points show that most of the variance in group size - cooperation association is attributable to the within-person effect, rejecting the possibility of the effect being driven purely by the between-level effect (*i.e.*, average prosocial tendency driving group size).

Effects of Partners' Faces

Some potential concerns might be raised to the confounding effect of particular faces on participants' behavior. Our dataset consisted of 30 neutral, ethnically diverse faces from the *NimStim* dataset.

Effects of partner's Race and Gender on Cooperation and Reciprocity.

- Between-person ANOVA on Cooperation rate as predicted by Gender and Race, revealed no significant effects of Gender $F(1,26)=1.045$ $p=0.366$, nor Race $F(2,26)=0.231$ $p=0.635$
- Between-person ANOVA on Reciprocity rate as predicted by Gender and Race, revealed no significant effects of Gender $F(1,26)=0.266$ $p=0.590$, nor Race $F(2,26)=0.539$ $p=0.610$.

Together, this suggests that, within the scope of the faces that were used for the experiment, the gender nor race of the partner did not influence behavior to a significant degree.

Variability in Cooperation and Reciprocity as a function of partner's face

- Cooperation levels based on partner's face varied from 0.459 (face number 5) to 0.677 (face number 1) with a mean of 0.573 and SD of 0.058
- Reciprocity levels based on partner's face varied from 0.625 (face number 22) to 0.757 (face number 4) with a mean of 0.699 and SD of 0.032
- The correlation between these two measures (how likely one is to cooperate with a given partner and how likely he is to reciprocate) were correlated at $r = 0.333$, $t(28) = 1.872$, $p=0.07$.

Effects of face memorability on behavior

If it was true that certain faces were remembered better than others, we should expect them to be associated with higher levels of reciprocity. Overall, the average reciprocity with different partners ranged from 0.625 (face number 22) to 0.757 (face number 4) with a mean of 0.699 and SD of 0.032. Additionally, we performed a regression analysis on pooled data, predicting reciprocity from individual faces. The model can be expressed symbolically as:

reciprocity ~ faceID

Here, *faceID* is a categorical factor variable with 30 different categories, corresponding to partners' faces. Significant effects would indicate that given face predicts reciprocity meaningfully better than the average.

The analysis did not provide significant coefficients for any of the faces. The largest positive coefficient was equal to 0.078 ($t=1.716$, $p=0.086$) for face 4. The largest negative coefficient was equal to -0.046 ($t=-1.031$, $p=0.302$) for face number 18. These results strongly suggest that face identity was not a significant predictor of reciprocity.

Supplementary References

1. Rand, D. G., Arbesman, S. & Christakis, N. A. Dynamic social networks promote cooperation in experiments with humans. *Proc. Natl. Acad. Sci.* **108**, 19193–19198 (2011).

2. Grujić, J., Eke, B., Cabrales, A., Cuesta, J. A. & Sánchez, A. Three is a crowd in iterated prisoner's dilemmas: experimental evidence on reciprocal behavior. *Sci. Rep.* **2**, 638 (2012).
3. Hamburger, H., Guyer, M. & Fox, J. Group size and cooperation. *J. Confl. Resolut.* **19**, 503–531 (1975).
4. Yamagishi, T. & Cook, K. S. Generalized Exchange and Social Dilemmas. *Soc. Psychol. Q.* **56**, 235–248 (1993).
5. Yamagishi, T. & Hayashi, N. Selective Play: Social Embeddedness of Social Dilemmas. in *Frontiers in Social Dilemmas Research* (eds. Liebrand, W. B. G. & Messick, D. M.) 363–384 (Springer, Berlin, Heidelberg, 1996). doi:10.1007/978-3-642-85261-9_19.
6. Rand, D. G., Nowak, M. A., Fowler, J. H. & Christakis, N. A. Static network structure can stabilize human cooperation. *Proc. Natl. Acad. Sci.* **111**, 17093–17098 (2014).
7. Bell, A., Jones, K. & Fairbrother, M. Understanding and misunderstanding group mean centering: a commentary on Kelley et al.'s dangerous practice. *Qual. Quant.* **52**, 2031–2036 (2018).
