# Supplementary material for: A neurocognitive mechanism for increased cooperation during group formation
Source: Commun Psychol. 2024 Dec 23;2:127. doi: 10.1038/s44271-024-00177-3 (PMC11666775; doi:10.1038/s44271-024-00177-3)
Supplement: Supplementary file 3 — Reporting Summary [file 44271_2024_177_MOESM3_ESM.pdf]

nature portfolio

Corresponding author(s): Wojciech Zajkowski, Rei Akaishi

Last updated by author(s): 2024/07/13

Reporting Summary

Nature Portfolio wishes to improve the reproducibility of the work that we publish. This form provides structure for consistency and transparency in reporting. For further information on Nature Portfolio policies, see our Editorial Policies and the Editorial Policy Checklist.

Statistics

For all statistical analyses, confirm that the following items are present in the figure legend, table legend, main text, or Methods section.

n/a	Confirmed	
☐	☒	The exact sample size (*n*) for each experimental group/condition, given as a discrete number and unit of measurement
☐	☒	A statement on whether measurements were taken from distinct samples or whether the same sample was measured repeatedly
☐	☒	The statistical test(s) used AND whether they are one- or two-sided<br>*Only common tests should be described solely by name; describe more complex techniques in the Methods section.*
☐	☒	A description of all covariates tested
☐	☒	A description of any assumptions or corrections, such as tests of normality and adjustment for multiple comparisons
☐	☒	A full description of the statistical parameters including central tendency (e.g. means) or other basic estimates (e.g. regression coefficient) AND variation (e.g. standard deviation) or associated estimates of uncertainty (e.g. confidence intervals)
☐	☒	For null hypothesis testing, the test statistic (e.g. *F*, *t*, *r*) with confidence intervals, effect sizes, degrees of freedom and *P* value noted<br>*Give P values as exact values whenever suitable.*
☐	☒	For Bayesian analysis, information on the choice of priors and Markov chain Monte Carlo settings
☐	☒	For hierarchical and complex designs, identification of the appropriate level for tests and full reporting of outcomes
☐	☒	Estimates of effect sizes (e.g. Cohen's *d*, Pearson's *r*), indicating how they were calculated

Our web collection on statistics for biologists contains articles on many of the points above.

Software and code

Policy information about availability of computer code

Data collection	MATLAB 2017a, Psychtoolbox
Data analysis	MATLAB 2021a, SPM 12, R 4.0.2, Stan Programming language

For manuscripts utilizing custom algorithms or software that are central to the research but not yet described in published literature, software must be made available to editors and reviewers. We strongly encourage code deposition in a community repository (e.g. GitHub). See the Nature Portfolio guidelines for submitting code & software for further information.

Data

Policy information about availability of data

All manuscripts must include a data availability statement. This statement should provide the following information, where applicable:

- Accession codes, unique identifiers, or web links for publicly available datasets
- A description of any restrictions on data availability
- For clinical datasets or third party data, please ensure that the statement adheres to our policy

The full behavioral data, analysis codes, and a video demonstration of the task are freely available at https://osf.io/45r3x

Human research participants

Policy information about studies involving human research participants and Sex and Gender in Research.

Reporting on sex and gender	Participants were asked to voluntarily report their biological sex in Japanese language according to standard self-report demographic questions in 2018 in Japan.
Population characteristics	For 25/83 subjects, their age was not recorded though all subjects were visibly young adult students (~20-30 years old). The mean age of the 62 subjects with recorded age information was 22.0 +/- 1.8 (SD).
Recruitment	Subjects were recruited through an online advertisement.
Ethics oversight	CiNet Research Ethics Committees, Osaka, Japan

Note that full information on the approval of the study protocol must also be provided in the manuscript.

Field-specific reporting

Please select the one below that is the best fit for your research. If you are not sure, read the appropriate sections before making your selection.

☐ Life sciences ☒ Behavioural & social sciences ☐ Ecological, evolutionary & environmental sciences

For a reference copy of the document with all sections, see nature.com/documents/nr-reporting-summary-flat.pdf

Behavioural & social sciences study design

All studies must disclose on these points even when the disclosure is negative.

Study description	Data come from a quantitative experimental design. The fMRI study involves imaging data from an approximately 30 minute fMRI scan, and behavioural data<br>during the fMRI scan and in a 30 minute post-scan session. The lesion patient study includes online behavioural data from 23 lesion patients and 27 age-matched healthy controls using the same task paradigm as in the fMRI study.
Research sample	Young adult Japanese volunteers, mostly University students.
Sampling strategy	Convenience sampling was used.<br>The full sample for assessing behavioral effects (83 participants) is significantly higher than the recommended sample of minimum 50 for finding within-person effects of averge effects size of Cohen's d = 0.4 with 80% power (which is considered a good first estimate of the smallest effect size of interest in psychological reseach):<br>Brysbaert M. How Many Participants Do We Have to Include in Properly Powered Experiments? A Tutorial of Power Analysis with Reference Tables. J Cogn. 2019 Jul 19;2(1):16.<br>Our fMRI sample of 26 was consistent (slightly higher) than the central tendency for fMRI studies involving healthy participants in top neuroimaging journals in recent years, which was equal to 24, according to:<br>Szucs D, Ioannidis JP. Sample size evolution in neuroimaging research: An evaluation of highly-cited studies (1990-2012) and of latest practices (2017-2018) in high-impact journals. Neuroimage. 2020
Data collection	The behavioral-only task was performed on a computer, in an experimental room, where an experimenter was present. The fMRI version was performed inside an MRI machine, with a computer monitor above the participant's head. Before each session, the subjects received the task instructions and watched a randomly chosen subject (in the current session) play approximately the first ten trials (up to the first stay/switch decision) as an experimenter-guided demo of the task. Subjects were told that their social partners could break network links with them, but subjects specifically were not instructed that maintaining network links was important to score or task performance. The timing of the start of the game play was synchronized so that each session groups' subjects could feel that they were playing the game together.
Timing	25/12/2018 - 22/01/2019
Data exclusions	4 participants (all participating in the behavioral-only session) were excluded from further analyses due to defecting on the vast majority (>95%) of trials. While we acknowledge that 'always defect' is a viable strategy used by a subset of population in prisoner's dilemma games, this a priori selection criterium served to remove participants who were completely unreceptive to the experimental manipulation. It is also very difficult to model subjects who always make the same choice, and would severely affect statistical sampling in the fMRI analysis to have little-to-no contrast in behavior.
Non-participation	No participants dropped out.
Randomization	Participants were not allocated into different groups; the experimental design was within-person.

Reporting for specific materials, systems and methods

We require information from authors about some types of materials, experimental systems and methods used in many studies. Here, indicate whether each material, system or method listed is relevant to your study. If you are not sure if a list item applies to your research, read the appropriate section before selecting a response.

Materials & experimental systems

n/a	Involved in the study
☒	☐ Antibodies
☒	☐ Eukaryotic cell lines
☒	☐ Palaeontology and archaeology
☒	☐ Animals and other organisms
☒	☐ Clinical data
☒	☐ Dual use research of concern

Methods

n/a	Involved in the study
☒	☐ ChIP-seq
☒	☐ Flow cytometry
☐	☒ MRI-based neuroimaging

Magnetic resonance imaging

Experimental design

Design type: event-related

Design specifications: A single block consisting of 180 trials. Each trial consisted of a PD choice (up to 3 s) and outcome (1.5s). Additionally, 10% of randomly chosen trials began with an introduction of a new partner (1s), and independently sampled 20% of trials ended with a stay/switch decision (up to 3s) followed by the outcome (2s). Trials were seperated by a intertrial interval sampled from a uniform distribution from 3 to 7s.

Behavioral performance measures: We measured: 1) binary PD choice (cooperate or defect) 2) PD choice reaction time 3) binary stay/switch choice (stay or switch) 4) stay/switch reaction times.

Acquisition

Imaging type(s): Functional and structural

Field strength: 3T

Sequence & imaging parameters: Echo-planar imaging (EPI) sequence with the following parameters: repetition time (TR)=2000ms, echo time (TE)=30 ms, flip angle=75°, field of view (FOV)=200 mm, slice thickness=2mm, voxel size = 2 × 2 × 2 mm3, multiband acceleration factor of 3, gap=0mm, ascending interleaved slice acquisition of 72 axial slices.

Area of acquisition: whole-brain

Diffusion MRI: ☐ Used ☒ Not used

Preprocessing

Preprocessing software: SPM 12

Normalization: The normalization method applied on all functional brain data was non-linear. We entered the subject specific T1 structural image as source image and the MNI standard T1 provided in the SPM12 toolbox as template image.

Normalization template: For each participant, the images were spatially normalized into the Montreal Neurological Institute (MNI) template, as implemented in SPM12 software.

Noise and artifact removal: To remove movement artifacts from the fMRI time-series, we realigned the functional brain images in SPM12 using default parameters. In the GLM, movement parameters were entered as nuisance regressors. We applied no artifact removal for respiration and heart rate.

Volume censoring: Not applied.

Statistical modeling & inference

Model type and settings: We used a mass-univariate approach. We constructed five GLM models to map model-based parameter values from the best fitting cognitive model to brain activity. All models had similar structure, such that the BOLD response in each voxel was predicted by the events (coded as stick regressors) related to: new partner introduction (10% of trials), choice, choice feedback, reward screen, switch (20% of the trials) and switch feedback (20% of trials). Additionally, all models contained parametric regressors of RT and memory decay parameter during choice period, a pause regressor (which reflected few-trial

long pauses affecting participants losing all their partners, until a new one is introduced), and 6 movement-related regressors.

Effect(s) tested

Standard higher-order statistical tests were performed on the group data estimates. GLM parameter estimates were first estimated at the level of run (first level), then combined within individuals as Fixed Effects

Specify type of analysis: ☐ Whole brain ☐ ROI-based ☒ Both

Anatomical location(s)

All ROIs were independently defined based on an online meta-analysis using keywords in the Neurosynch database (http:// neurosynth.org). The keywords were dictated by the hypothesized function of these areas within the context of our task. In order to obtain a reasonable sample size of studies, the keywords were as general as possible. Additionally, the activity clusters needed to be located within the anatomical bounds of the region for which the ROI was being obtained. All ROIs were spherical, with varying volumes. The volume of the ROIs were dictated by a) anatomical size of the structure of interest, and b) the size of the functional cluster, within the arbitrary limits of not being smaller than 5 mm3, and not larger than 10 mm3. ROI analysis was performed by extracting whitened and filtered voxels within an ROI from a contrast of interest, and interest and averaging the voxel values within participants. The vector of averaged beta weights was then compared against 0 using a t-test.
Value of cooperation. The VMPFC ROI was defined as 10 mm3 sphere, centered at the peak activity for the keyword 'value' (peak MNI coordinates: x=0, y=40, z=-8; 407 studies included in the database). Choice of VMPFC was driven by the a priori expectation of the region being involved in subjective value processing.
Forgetting. This set of ROI was chosen in order to test the connectivity between memory and value representations, and included: precunes, FFG, NAcc and dACC.
The precuneus ROI was defined as 10 mm3 sphere, centered at the peak activity for the keyword 'memory' (peak MNI coordinates: x=-8, y=-66, z=28; 2744 studies included in the database). Choice of precuneus was motivated by both: an a priori expectation of the region playing a crucial role in social memory as well as the functional results of earlier analyses, relating its activity to forgetting.
The FFG ROI was defined as two 6 mm3 spheres, centered at the peak activity for the keyword 'face recognition' (peak MNI left: x=-42, y=-52, z=-20; peak MNI right: x=41, y=-52, z=-20; 79 studies included in the database). The choice of FFG was motivated by both: an a priori expectation of the region being involved in face recognition and memory, as well as the functional results of earlier analyses, relating its activity to forgetting.
The NAcc ROI was defined as two 5 mm3 spheres, centered at the peak activity for the keyword 'value' (peak MNI left: x=-6, y=10, z=-5; peak MNI right: x=6, y=-10, z=-5; 407 studies included in the database). The choice of NAcc was motivated by both: an a priori expectation of the region being involved in reward processing, as well as the functional results of earlier analyses, relating its activity to the value of cooperation.
The dACC ROI was defined as a 10mm3 sphere centered at the peak activity for the keyword 'control' (peak MNI: x=4, y=18, z=42; 3796 studies included in the database). The choice dACC was motivated by both: an a priori expectation of the region being involved in integrating value information across timescales, as well as the functional results of earlier analyses, relating its activity to the value of reciprocity.
Congruence. For the congruence analysis, we re-used the precuneus and dACC ROIs defined above. Additionally, we built 2 ROIs for the left and right DLPFC, defined as 5 mm3 spheres, centered at [-40, 40 24] and [40, 40 24], respectively. Similarly, to the earlier definition of dACC, we used Neurosynch peak coordinates related to the term 'control', within clusters which anatomically included the DLPFC region. We chose those regions for both theoretical and practical reasons (i.e., significant whole-brain activity related to key functional roles in prior GLM models). DLPFC and dACC have been implicated in representing value-based information across different timescales, while the precuneus is a central hub of the mentalizing network, suggested to play a role in social working memory.

Statistic type for inference
(See Eklund et al. 2016)

All reported effects are whole brain family-wise error (FWE) cluster level $P < 0.05$ corrected, after thresholding at $P < .001$ uncorrected.

Correction

$P < 0.05$ cluster-wise FWE corrected, with cluster-forming threshold $P < 0.001$ uncorrected.

Models & analysis

n/a	Involved in the study
☐	☒ Functional and/or effective connectivity
☒	☐ Graph analysis
☒	☐ Multivariate modeling or predictive analysis

Functional and/or effective connectivity

We performed an ROI-based PPI analysis to test the connectivity between a) precuneus and fusiform gyri, and b) precuneus and NAcc, and c) precuneus and dACC during the choice period, as a function of memory retention . The psychophysiological interaction (PPI) analysis was performed using the gPPI (generalized PPI) toolbox (Maclaren et al. ,2012)which explicitly models the whole task on top of the PPI-specific regressors. We tested our hypotheses by testing the connectivity of the seed with a) fusiform, b) NAcc, and c) dACC, as a function of the memory retention regressor.
